# The mediating role of default mode network during meaning-making aroused by mental simulation between stressful events and stress-related growth: a task fMRI study

**DOI:** 10.1186/s12993-023-00214-x

**Published:** 2023-07-15

**Authors:** Yidi Chen, Jinjin Ma, Huanya Zhu, Huini Peng, Yiqun Gan

**Affiliations:** School of Psychological Cognitive Sciences, Beijing Key Laboratory of Behavior and Mental Health, Peking University, Beijing, 100871 China

**Keywords:** Stressful events, Default mode network, Meaning-making, Stress-related growth, Psychophysiological interaction

## Abstract

**Background:**

Stressful events and meaning-making toward them play an important role in adolescents’ life and growth. However, ignoring positive stressful events leads to negativity bias; further, the neural mechanisms of meaning-making are unclear. We aimed to verify the mediating role of meaning-making in stressful events and stress-related growth and the function of the default mode network (DMN) during meaning-making in this functional magnetic resonance imaging (fMRI) study.

**Methods:**

Participants comprised 59 university students. Stressful life events, meaning-making, and stress-related growth were assessed at baseline, followed by fMRI scanning during a meaning-making task aroused by mental simulation. General linear modeling and psychophysiological interaction (PPI) analyses were used to explore the activation and functional connectivity of DMN during meaning-making.

**Results:**

Mental simulation triggered meaning-making, and DMN activity decreased during meaning-making. Activation of the DMN was negatively correlated with coping flexibility, an indicator of stress-related growth. PPI analysis showed that meaning-making was accompanied by diminished connectivity in the DMN. DMN activation during meaning-making can mediate the relationship between positive stressful events and coping flexibility.

**Conclusions:**

Decreased DMN activity and diminished functional connectivity in the DMN occurred during meaning-making. Activation of the DMN during meaning-making could mediate the relationship between positive stressful events and stress-related growth, which provides a cognitive neural basis for the mediating role of meaning-making in the relationship between stressful events and indicators of stress-related growth.

**Implications:**

This study supports the idea that prosperity makes heroes, expands the meaning-making model, and suggests the inclusion of enhancing personal resources and meaning-making in education. This study was the first to validate the activation pattern and functional connectivity of the DMN during meaning-making aroused by mental simulation using an fMRI task-state examination, which can enhance our sense of meaning and provide knowledge that can be used in clinical psychology interventions.

***Trial Registration*:**

The study protocol was pre-registered in Open Science Framework (see osf.io/ahm6e for details).

**Supplementary Information:**

The online version contains supplementary material available at 10.1186/s12993-023-00214-x.

## Background

There is an old saying, “What does not kill us makes us stronger” [1]. It describes the role of adversity in personal growth, which has been eulogized by the pioneers. Others have emphasized the role of good fortune in personal growth, with their thoughts represented by the old saying, “The good wind helps me go up into the clouds.” The debate over the role of good times and bad times has never stopped, and it continues in psychology with the study of stressful events [2]. For a long time, researchers have associated stress with negative effects, assuming stress inevitably leads to negative outcomes such as anxiety, depression, or impaired social adjustment [3].

However, a growing body of research also suggests that stress does not necessarily lead to negative outcomes for individuals [4]. In contrast, studies show that effective stress coping leads individuals to perceive a sense of control and to better cope with subsequent stressful events [5] and that some stress may even have positive effects [6]. Recent research suggests that approximately two-thirds of individuals who experience stressful events or adversity concomitantly demonstrate adaptive functioning, exhibiting what is commonly referred to as “stress resilience” despite the severity and persistence of the related life event experience [7].

Individual differences after experiencing stress have attracted the attention of the academic community and even triggered a paradigm shift in stress, clinical psychology, and psychiatric research [8]. The paradigm shift refers to the current, and ample, evidence that all people—not just those who are supposedly susceptible to stressors—undergo changes in response to stressors; such evidence makes it important to explore the coping strategies and cognitive processes that occur during these responses.

We believe that cognitive neuroscience methodologies could offer new perspective into this process. Specifically, they enable the delineation of the physiological basis of post-stress disorder or adaptation in terms of the ways in which brain activity modulates stress-coping processes and triggers different coping outcomes.

## Meaning-making strategy promotes stress-related growth

Stress-related growth (SRG), a type of personal growth after stressful life events, mainly manifests through enhanced social and personal resources and developed or changed coping skills [9]. Previous studies observed that coping flexibility is associated with enhanced performance under stress [10]. Coping flexibility refers to the ability to discontinue ineffective coping strategies and generate and implement alternative coping strategies [11], which could help individuals shift their perspectives and ways of thinking, generate new strategies, and cope better with stress. SRG occurs when individuals extract meaning from the potential negative effects of stressful events. This process requires both the ability to switch cognitive subsets and the ability to evaluate and switch strategies in coping. Therefore, for SRG to occur and for one’s perceptions toward stressful events to change, mindset and strategy shifts become critical [12]. Individuals with high coping flexibility were more likely to change their schemas in a positive direction, compared with those with low coping flexibility [9]. Further, individuals with high coping flexibility make greater use of adaptive coping strategies in stressful situations, with the utilization of multiple adaptive coping strategies being associated with better resilience and fewer mental health problems [10, 11]. Thus, coping flexibility has been used as an indicator of SRG.

The cyclical model of stress resilience and SRG assumes that meaning-making is an effective strategy to facilitate an individual’s ability to create positive outcomes [9]. Meaning-making refers to an individual’s adaptive function under stress and the process by which they reconcile their beliefs and goals to cope with stressful situations, thus changing the way they assess a situation [6, 12]. The meaning-making model suggests that individuals possess both global and situational meanings; when individuals are faced with negative stressful situations, these global and situational meanings can conflict with each other and cause distress. Thereafter, individuals spontaneously generate meaning-making processes that result in outcomes such as the individual’s perceived SRG and positive changes. For this process, negative stressful situations are a prerequisite, and differences in situational and global meanings are the driving force behind individuals’ meaning-making [12].

The meaning-searching process can be exemplified through the process of accumulating individual autobiographical memories throughout one’s life course, with individuals integrating temporal information to construct meaning to guide their future lives [12]. This process involves some narrative components that are used by individuals to find common threads in past, present, and future experiences. These common threads enable them to create a coherent cognitive representation that triggers SRG and predicts higher psychological well-being [12]. Both nostalgia and thinking about the future trigger meaning-making processes that enhance self-reported sense of meaning in life and positive emotions [13, 14]. Moreover, mental simulation allows for individuals to leave the “here and now” through self-alienation—a process that integrates temporal information and the gain of causal apprehension, serving as a stable paradigm for triggering meaning-making [15–17]. A recent study on mental simulations found that imagining two separate events that take place in the future can initiate meaning-making processes and result in more making in life. However, imagining two separate events in the present does not; therefore, in this study, a comparison of future and present mental simulations was used to characterize meaning-making [17].

The cyclical model of stress resilience and SRG suggests that coping strategies, such as meaning-making, can act as mediators that lead to SRG: namely, meaning-making can mediate the relationship between stress and SRG. The meaning-making model also proposes that negative stressful events provide the premise for meaning-making and facilitate meaning-making in response to stress, which triggers SRG [9].

### Negativity bias in the field of stress-related growth

The meaning-making model suggests that negative stressful events trigger meaning-making and SRG [12]. Research in the field of psychological growth has typically conducted between-group comparisons regarding the mental health of individuals experiencing low, moderate, and high levels of stressful events. Findings suggest that very low levels of stress result in little growth compared with moderate levels of stress [18]; further, the presence of post-traumatic growth suggests that individuals experiencing high levels of stress also show growth [19]. However, these findings coincide with researchers’ beliefs that negative stressful events lead to the destruction of our basic beliefs, to the reconstruction of coping and core beliefs, and to the generation of SRG [12]; that is, they exclude the role of positive life stress, leading to a negativity bias in the field of psychological growth.

In recent times, however, researchers have started to focus on the role of positive stressors in promoting growth [20]. In fact, a meta-analysis of prospective studies showed that both negative and positive stressful events were followed by positive trends in the subdomains of self-esteem, positive relationships, and personal growth [21]. Researchers have hypothesized that any unusual event, regardless of its valence, can change core beliefs, and that positive experiences also trigger meaning construction and can act as catalysts for growth [22].

### The function of default mode network in meaning-making

Despite the evidence on meaning-making promoting SRG, no research has explored the cognitive neural mechanisms underlying this process [12]. In the affective neuroscience model of boosting resilience, three effective pathways exist for maintaining stress resilience during stressful situations: increasing positivity, decreasing negativity, and transcending the self. These correspond to three distinct brain circuits: the reward network, amygdala, and DMN, respectively [23]. Meaning-making is an important coping strategy for transcending the self, and the latter is represented by DMN activity. Specifically, by reducing the activation of the DMN associated with self-reflection and rumination or by meaning-making, individuals can have experiences that promote self-transcendence; such promotion helps them reflect on long-term meaning-making related to stress, thus facilitating SRG [24, 25].

The DMN network is the most important subnetwork, being characterized by reduced activity in active attention-demanding tasks. Specifically, it comprises the medial prefrontal cortex (mPFC), posterior cingulate cortex (PCC), precuneus, inferior parietal lobe, hippocampus, inferior temporal cortex, and several other brain regions [15]. The DMN function is involved in attention to external and internal stimuli and in self-referential and reflective activities [26].

Early stressful life events are associated with connectivity patterns within the DMN during rest in early adulthood. Exposure to life stress during infancy might have long-lasting influences on functional brain connectivity that persist until early adulthood [27]. Therefore, the DMN plays an important role in the regulation of mental health under stress. Longitudinal fMRI studies in adolescents have found that subclinical depression and post-traumatic symptoms alter the trajectory of DMN connections, which may indicate that the network is a clinically considerable link in mental health disorders [28]. The dynamic model of thought-roaming suggests that overactivity of the DMN (including the mPFC and PCC) in the default network core leads to automatic constraints on thought increase; this leads to rumination and obsessive thinking, which are characteristic of mood and anxiety disorders [25]. Concurrently, DMN hyperactivity is associated with psychiatric disorders, such as depression and schizophrenia [29]. When engaging in self-reference thinking, individuals who meditate frequently show greater deactivation of the mPFC and PCC than individuals in the control group [30]. Thus, being able to reduce DMN hyperactivity and disengage from rumination may contribute to one’s stress resilience [23, 30, 31].

The literature also shows that an abnormal functional connectivity (FC) of the DMN contributes to stress-related disorders. For example, resting-state studies with patients with depression showed elevated intrinsic connectivity of the DMN [32], and meta-analyses have shown an association between depression and enhanced intrinsic positive connectivity, as well as between the first and diminished negative connectivity, of the DMN [33].

Despite these pieces of evidence, the reality is that findings remain inconsistent regarding the relationship between DMN connectivity and mental health. For example, in a large sample of resting-state studies, patients with depression showed reduced DMN FC compared with healthy controls [34]. This contrasts with the results of the previously cited research.

However, the evidence primarily related to the relationship between the DMN and stress-related disorders. Regarding meaning-making, only one study has linked it to the DMN [17]; in this study, enhanced connectivity of the medial temporal network, which is a subnetwork of the DMN, was associated with a self-reported meaning in life on a resting-state MRI. In other words, the study provides support for the regulatory role of DMN in meaning-making [17].

### The present study

Stress is generally associated with maladjustment and dysfunction [1]; however, some studies have shown that stress can have positive effects and produce SRG [1, 4, 5]. The meaning-making model suggests that meaning-making is effective in helping individuals grow after stressful experiences [12, 19]. The affective neuroscience model of boosting resilience indicates that meaning-making is a strategy, mediated through the DMN, to transcend self to acquire coping skills and achieve SRG [23].

Despite the invaluable contributions of existing research, the studies have some limitations: first, they have mostly focused on meaning-making from negative stressful events, neglecting positive ones [20, 21]. Second, although previous research has identified the role of meaning-making in stress adaptation [6, 12], few have used a laboratory context to assess how the cognitive neural mechanisms of meaning-making contribute to SRG and coping flexibility in daily life. Third, the relationships between hyperactivity in the DMN and rumination and between the first and maladaptation under stress have been validated [25]; however, only one resting-state study has demonstrated the cognitive neural mechanisms of meaning-making [15]. To date, no studies have explored the activity and FC of the DMN during meaning-making; thus, the relationship between activity and FC of the DMN and SRG requires elucidation.

Following prior research [17], we used the paradigm of mental simulation triggering meaning-making to observe DMN activity and FC during meaning-making in a task fMRI. Specifically, whole-brain activation analysis and regions of interest (ROI) analysis were used to explore the decline in DMN activity during meaning-making and its association with SRG. Psychophysiological interaction (PPI) analysis was used to explore the diminished FC within the DMN under meaning-making using the PCC core seed point of the DMN as a starting point. The PCC, as a core subregion of the DMN, was often selected as the starting point for PPI [35–37]. We also built mediation models to explore whether DMN activation during meaning-making can mediate the pathway from different stressful events to the SRG. The exploratory mediation model was validated to determine whether good times or bad times are conducive to personal growth.

## Results

### Manipulation check of mental simulation on meaning-making

Participants rated their meaning-making scores and meaning-in-life scores after they completed their mental simulation task. We used a repeated-measures multivariate analysis of variance to assess whether significant differences existed in participants’ meaning-making and meaning-in-life scores after they completed their meaning-making and control conditions.

The results indicated that the main effect of time condition was significant [*F*(2, 57) = 5.39, *p* = .007, partial *eta*^2^ = 0.16]. Participants reported more meaning-making (*M*_meaning−making_ = 3.58, *SE* = 0.08; *M*_control_ = 3.49, *SE* = 0.08) and a higher meaning in life (*M*_meaning−making_ = 3.67, *SE* = 0.06; *M*_control_ = 3.52, *SE* = 0.07) after the meaning-making condition compared with the control condition.

### Correlations between self-reported measures

To control for the type I error inflation caused by multiple comparisons, the significance level (α) of the correlation analyses was adjusted to 0.0125 with Bonferroni correction [38]. Previous studies have demonstrated that sex, age, and education are significantly associated with SRG [39–41] and meaning-making [42–44]. After we controlled for participants’ sex, age, and education, the questionnaire results indicated significant positive correlations between meaning-making and the scores for SRG (*r* = .53, *p* < .001) and coping flexibility (*r* = .60, *p* < .001). The scores for SRG were significantly positively correlated with coping flexibility (*r* = .51, *p* < .001); however, the scores for positive stressful events were not significantly positively correlated with coping flexibility (*r* = .27, *p* = .043).

### Generalized linear modeling analysis

**Fig. 1:**
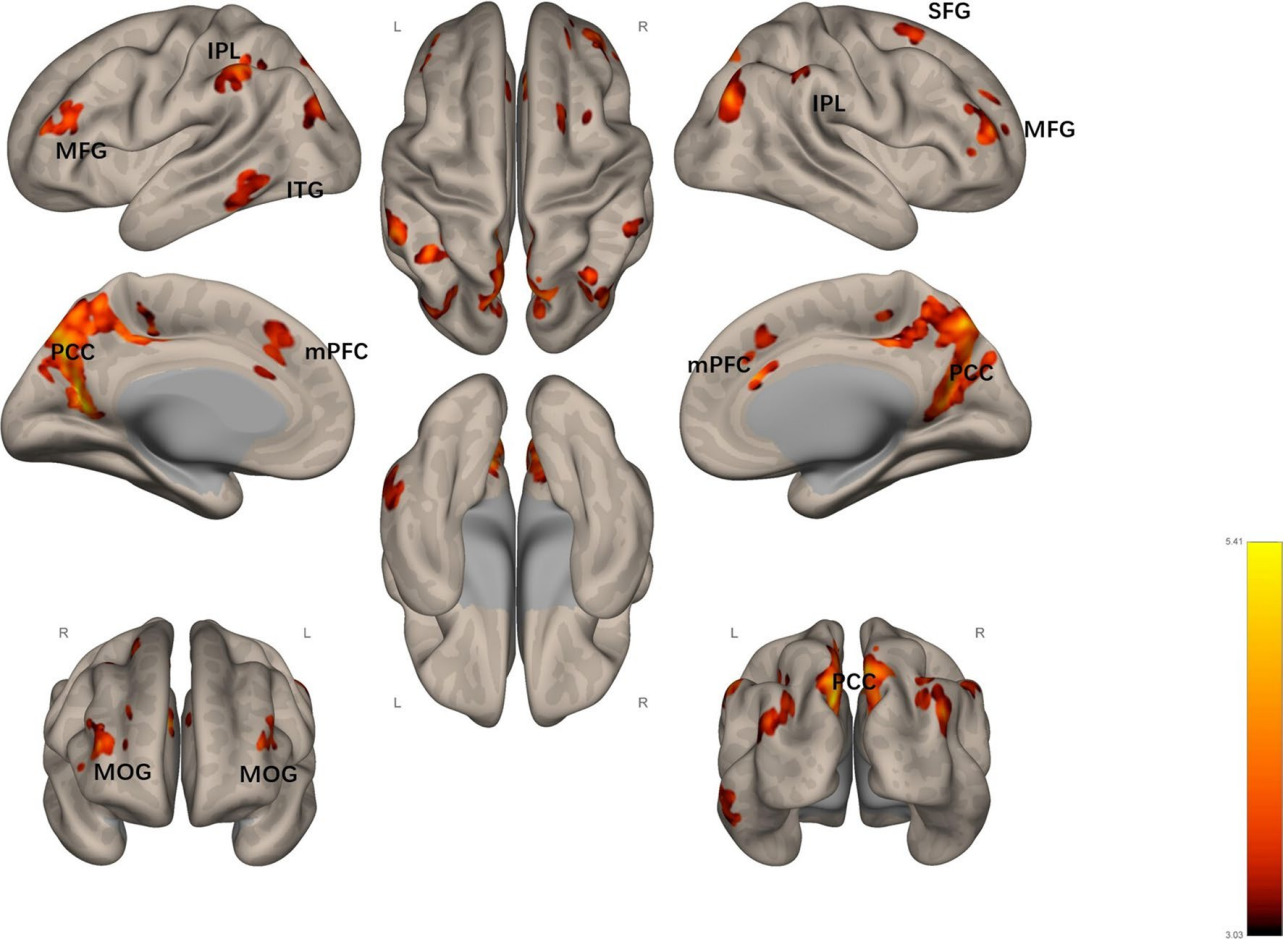
Negative activation of brain regions under meaning-making > control contrast (colored) L represents Left and R represents Right. *IPL* inferior parietal lobule, *ITG* inferior temporal gyrus, *MFG* middle frontal gyrus, *mPFC* medial prefrontal cortex, *MOG* middle occipital gyrus, *PCC* posterior cingulate cortex, *SFG* superior frontal gyrus

Through a Generalized Linear Modeling (GLM) analysis, we detected the brain areas associated with meaning-making since we compared the meaning-making and control conditions (contrast: meaning-making > control). Participants reported higher meaning-making and meaning-in-life scores under the meaning-making condition than under the control condition. Thus, the activation of the brain areas in the meaning-making > control condition could be considered an active process of meaning-making. In the meaning-making > control condition, no positive brain area activation was observed (*p* < .05, false discovery rate (FDR) corrected at voxel level; extent threshold k > 64). However, negative brain area activation was observed in the following areas: the left precuneus, right cuneus, bilateral middle frontal gyrus, bilateral middle occipital gyrus, bilateral inferior parietal lobe, right superior frontal gyrus, and left inferior temporal gyrus (Table 1; Fig. 1); this result indicated a decrease in the core system of DMN: the PCC, the mPFC, and the temporal lobe subsystems.

**Table 1:** Negative activation of brain regions under meaning-making > control contrast

Region label	Extent	t-value	MNI Coordinates		
			x	y	z
Meaning-making (present > future)					
L Precuneus	2526	6.31	− 10	− 68	38
R Cuneus	2526	5.94	18	− 60	26
R Middle frontal gyrus	92	5.25	46	50	10
R Middle occipital gyrus	234	5.24	42	− 76	36
L Inferior parietal lobule	122	4.98	− 56	− 40	44
L Middle frontal gyrus	120	4.89	− 42	48	18
mPFC	249	4.77	2	32	26
R Superior frontal gyrus	100	4.52	36	56	16
R Middle frontal gyrus	77	4.40	26	16	62
R Inferior parietal lobule	64	4.29	54	− 34	58
L Middle occipital gyrus	113	4.24	− 30	− 74	32
L Inferior temporal gyrus	100	4.08	− 60	− 48	− 16

*p* < .05, FDR corrected at voxel level; extent threshold k > 64

### Regions of interest analysis

Using the DMN and on the basis of the brain network of the functional regions segmented by Yeo et al. [60] as the ROI, we extracted the mean activation values of the DMN in the meaning-making > control condition. To control for the type I error inflation caused by multiple comparisons, the significance level (α) of the correlation analysis was adjusted to 0.025 with Bonferroni correction. After we controlled for participants’ sex, age, and education, we observed that positive stressful events were significantly negatively correlated with DMN activation (*r* = − .39, *p* = .004). Further, coping flexibility was also significantly negatively correlated with activation of the DMN (*r* = − .37, *p* = .005).

After we controlled for sex, age, and education, we observed that DMN activation in the meaning-making > control condition was not significantly correlated with negative stressful events (*p* > .05).

### Mediating effect analysis

We used the activation of the DMN (to serve as the cognitive neural indicator of meaning-making) as a mediating variable, SRG as the outcome variable, and negative and positive stressful events as independent variables in separate analyses. To control for the type I error inflation caused by multiple comparisons, the significance level (α) of the mediating analyses was adjusted to 0.025 with Bonferroni correction [97.5% confidence interval (CI)]. We found that the mediating effect was not significant in either the positive or the negative stressful event (negative stressful events: *B* = 0.01, *β* = 0.01, *SE* = 0.03, 95% CI [− 0.04, 0.07]; positive stressful events: *B* = − 0.02, *β* = − 0.02, *SE* = 0.07, 95% CI [− 0.17, 0.10]). Since the 95% CI included zero, the 97.5% confidence interval definitely included zero; thus, the mediating effect was nonsignificant.

However, only one mediating model was established between positive stress events and coping flexibility; thus, the significance level (α) of the mediating analyses was chosen as 0.05 (95% CI). On controlling for sex, age, and education, we observed a significant indirect effect of DMN activation (i.e., a cognitive neural indicator of meaning-making) in the relationship between positive stressful events and coping flexibility [*B* = 0.08, *β* = 0.12, *SE* = 0.08, 95% CI (0.01, 0.32)]. However, the direct effect was nonsignificant [*B* = 0.11, *β* = 0.17, *SE* = 0.15, *t* = 1.17, *p* = .246, 95% CI (− 0.12, 0.47)].

### Psychophysiological interaction analysis

**Fig. 2:**
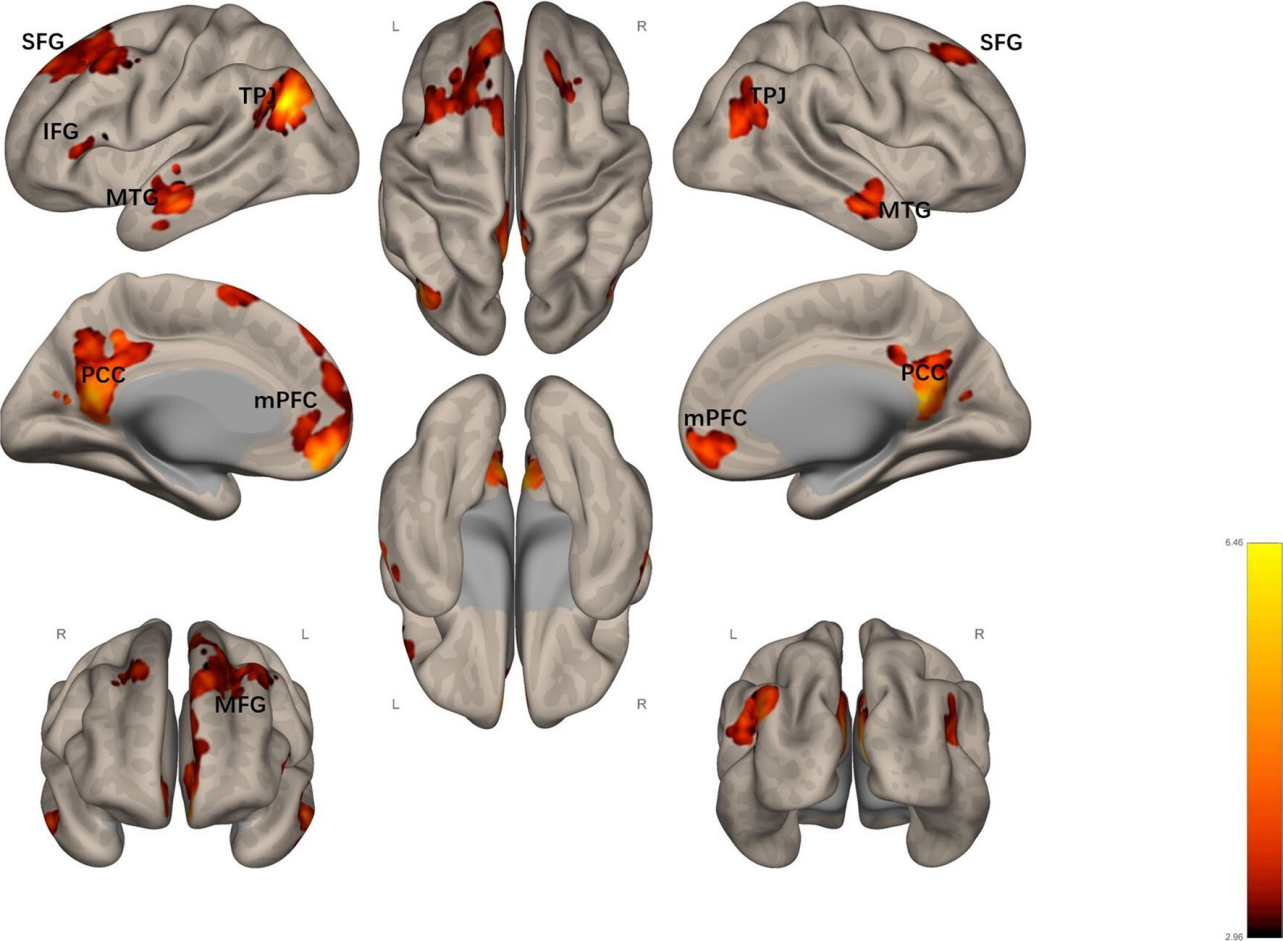
Brain regions with significantly reduced connectivity to PCC under meaning-making conditions (meaning-making > control contrast; colored) L represents Left and R represents Right. *IFG* inferior frontal gyrus, *MFG* middle frontal gyrus, *mPFC* medial prefrontal cortex, *MTG* middle temporal gyrus, *SFG* superior frontal gyrus, *PCC* posterior cingulate cortex, *TPJ* tempo-parietal junction

ROI analysis revealed that activation within the DMN declined during meaning-making. Therefore, we used PPI analysis to explore FC within the DMN during meaning-making. The PPI analysis revealed that, in meaning-making conditions, the seed point of meaning-making (PCC, − 10 − 68 38) showed significantly reduced FC of the PCC and other core subsystems of the DMN compared with the control conditions (*p* < .05, FDR corrected at voxel level; extent threshold k > 64) in the following regions: the bilateral angular gyrus, left middle cingulate gyrus, right precuneus, right calcarine gyrus, bilateral middle temporal gyrus, left rectus, bilateral superior frontal gyrus, left middle frontal gyrus, left posterior medial frontal, and left inferior frontal gyrus of the orbit (Table 2; Fig. 2).

**Table 2:** Brain regions with significantly reduced connectivity to PCC under meaning-making conditions (meaning-making > control contrast)

Region label	Extent	t-value	MNI coordinates		
			x	y	z
Meaning making (future < present)					
L Tempo-Parietal Junction	714	7.65	− 48	− 70	32
L Precuneus	1325	6.69	− 6	− 46	40
R Precuneus	1325	6.30	6	− 52	12
L Middle temporal gyrus	369	6.12	− 56	− 8	− 16
R mPFC	595	5.15	10	58	− 10
R Middle temporal gyrus	242	5.21	58	− 2	− 18
R Superior frontal gyrus	171	4.99	22	38	48
L Middle frontal gyrus	659	4.83	− 36	14	58
L Superior frontal gyrus	659	4.76	− 12	46	44
R Tempo-parietal junction	274	4.73	48	− 66	30
L mPFC	98	4.66	− 12	10	72
L Inferior frontal gyrus	69	4.19	− 52	24	0

*p* < .05, FDR corrected at voxel level; extent threshold k > 64

## Discussion

This study demonstrated a significant correlation between meaning-making and SRG and decreased DMN activity during meaning-making. Consistent with our hypothesis, we identified a significant negative correlation between DMN activation and coping flexibility (an indicator of SRG). Meaning-making was accompanied by diminished FC between PCC and mPFC, as well as between the temporal lobe subsystems. Further, DMN activation during meaning-making could mediate the relationship between positive stressful events and coping flexibility.

### Role of the default mode network in meaning-making

This study was the first to validate the activation pattern and FC of the DMN during meaning-making aroused by mental simulation using an fMRI task-state examination. Consistent with previous studies, considering the future or the past activates the meaning-making system [45]. Imagining the future can spontaneously produce meaningful events whether one is asked to imagine specific events or is not given additional cues. In particular, when two separate events are asked to be imagined separately, the process of meaning construction is enhanced by the causal connection of future events [17].

We compared two separate events related to imagining the future with two separate events related to imagining the present and found that the DMN was less activated when imagining the future with two separate events, in which the participants engaged in meaning-making. Since imagination occurred in both the meaning-making and control conditions, this diminished activation can be inferred to have been triggered by the meaning-making caused by future mental simulation. Compared with the control condition, the following DMN regions showed a lower activation during the meaning-making condition: the left precuneus, right cuneus, bilateral middle frontal gyrus, bilateral middle occipital gyrus, bilateral inferior parietal lobe, right superior frontal gyrus, and left inferior temporal gyrus.

Consistent with previous studies, decreased activation of these areas indicates stress adaptation and improved mental health. In the left precuneus and right cuneus, as the core region of DMN, inactivation was associated with recovery from depressive mood [46]. Increased activation of the middle frontal gyrus, which is a part of the dorsolateral PFC, is associated with decreased efficiency of working memory and increased risk for certain genotypes of schizophrenia [47]. Superior frontal gyrus is a core brain region for cognitive control and emotion regulation-related processes. Increased activation of the right superior frontal gyrus correlates with stress perception; therefore, decreased activation in this region could predict better stress adaptation [48]. The inferior parietal lobe serves as a major hub for integrating multisensory information inputs for comprehension and manipulation [49]. It is also an important component of the DMN [29]. An fMRI study conducted among 205 previously deployed U.S. military veterans showed that the left IPL alone was positively correlated with PTSD symptom severity [50]. Early life stress and psychological stress are positively correlated with regional activity in the inferior temporal gyrus [51, 52]. Hence, activity in the DMN decreased during increased meaning-making, which delineated a cognitive neural basis for meaning-making and its promotion of SRG. This evidence partially validated previous research on rumination: indulging in ruminative reflections of the self in the here and now was associated with DMN hyperactivity [23], which triggered poor emotional management or stress-related disorders. In contrast, mental simulations related to the future lead to processes that are antithetical to ruminative thinking, being accompanied by decreased DMN activity.

The results also provide evidence for the relationship between the FC of the DMN and meaning-making. Previous research has shown that sense of meaning and the subnetwork connectivity of the DMN are positively correlated [17]. However, we demonstrated that the FC of the DMN decreased during meaning-making. Specifically, the results of the PPI suggested that FC within the DMN, between the PCC and mPFC, as well as the temporal lobe subsystems, was diminished during meaning-making.

These results were corroborated, at least to an extent, by the relationship between DMN hyperactivation and psychological disorders. For example, some depressive symptoms were mediated by high intrinsic connectivity of the DMN [32]. Moreover, a study showed that the diminished connection between the PCC and mPFC could serve as a strategy for effective stress coping and avoiding the development of mood disorders, such as depression and anxiety [30]. Therefore, the weak FC of the PCC and mPFC can provide the basis for SRG.

Moreover, DMN activation and coping flexibility were negatively correlated, and DMN hyperactivation during meaning-making was detrimental to SRG. This was evidenced by decreased coping flexibility. When coping with stress, coping flexibility facilitates the formation of new core beliefs, and individuals with high coping flexibility tend to evaluate stressful situations and generate alternative solutions instead of indulging in rumination, promoting SRG [9, 11]. The relationship between coping flexibility and decreased DMN activity during meaning-making provides an explanatory neural pathway for meaning-making and its role as a facilitator of SRG.

### Extension of the meaning-making model by positive stressful events

Consistent with our hypothesis, our findings suggest that meaning-making can mediate the relationship between positive stressful events and SRG, highlighting the role of positive stressful events on SRG. Hence, SRG arises when individuals make meaning during positive stressful events, not only during negative stressful events. A previous study of autobiographical memory showed that positive recall facilitated meaning-making by evoking positive emotions and allowed individuals to discover the meaningful components of the event [22]. Further, positive stressful events can serve as turning points that enhance individuals’ stress resilience [21]. The findings also validate Seligman’s PERMA model, which emphasizes the importance of positive affect and meaning-making for growth. In this model, for situational meanings involving specific events, the meanings made for positive events can be associated with a better self and allow for self-transcendence and growth [53].

In addition, our research demonstrated that DMN activation under meaning-making can mediate the relationship between positive stressful events and coping flexibility. This serves as neurophysiological evidence for the debate on good times versus bad times in stress research [21]. Individuals who experience more positive stress have decreased DMN activity during meaning-making, which can have a mediating effect on coping flexibility. However, we did not verify the neurophysiological relation between negative stressful events and SRG because college students may experience fewer negative stressful events compared with the large-scale national sample of a previous study [1].

### Theoretical implications

First, this study is the first to use task-state fMRI to explore the cognitive neural mechanisms, as well as the activation and FC of the DMN, during meaning-making aroused by mental simulation. This study will pave the way for groundbreaking research in the field of cognitive neuroscience of meaning-making. Individuals will have lower DMN activity during meaning-making after having experienced numerous positive stressful events, which will lead to enhanced coping flexibility during stressful events, and thus SRG. This provides a cognitive neural basis for the mediating role of meaning-making in the relationship between stressful events and indicators of SRG.

Second, this study extends the applicability of the meaning-making model [12]. In addition to traumatic and negative stressful events, we demonstrated that positive stressful events could change an individual’s core beliefs and trigger SRG, an outcome of meaning-making. Therefore, positive stressful events are also important factors for SRG research, abating the negativity bias in the research domain of cumulative stressful events.

### Practical implications

This study validated the effectiveness of mental simulation activities related to future events for meaning-making, which can enhance our sense of meaning and provide knowledge that can be used for interventions in clinical psychology. Future research can stimulate meaning-making in individuals through mental simulation or self-alienation interventions [16, 17]. These initiatives may enhance SRG and stress-coping abilities. Our results also provide a practical basis for future use of non-invasive brain stimulation-based interventions (e.g., DCTs and TMS) to enhance mental health during stressful situations [54, 55].

### Limitations and future research directions

This study has some limitations. First, we used only mental simulation as the scanning task, a well-established paradigm for triggering meaning-making. Although we observed elevated meaning-making in the meaning-making > control condition, we observed correlations only between the meaning-making process and the FC and DMN activation. Future research should use other paradigms (e.g., meaning-making in a stressful situation) to observe more direct meaning-making processes [12].

Second, the study was conducted with a set of imagery as a factor in the GLM. However, we could not observe how the regions in the DMN interacted with each other during meaning-making. This is an avenue for future research.

Third, this study investigates the role of positive and negative life events in meaning-making separately, expanding the traditional meaning-making model. However, in real life, the effects of positive and negative events are not independent but interact with each other [2], which warrants further exploration. A longitudinal design could be used to assess whether positive and negative stressful events can interact to influence individuals’ meaning-making and growth.

Finally, the study was theoretically driven to verify the mediating role of the DMN on the relationship between stressful events and SRG during the meaning-making process. Future research should explore the relationship among the indicators of executive control, flexibility, and FC among different brain networks and their predictions on SRG.

## Conclusions

Decreased DMN activity and diminished FC in the DMN occurred during meaning-making. Activation of the DMN during meaning-making could mediate the relationship between positive stressful events and SRG, which provides a cognitive neural basis for the mediating role of meaning-making in the relationship between stressful events and indicators of SRG.

## Methods

### Participants

A sample of 60 adult college students (30 women and 30 men) were recruited from China. The sample size was determined on the basis of previous fMRI studies [17]. However, one participant failed to complete the fMRI owing to excessive head movements. Thus, 59 participants were included in the follow-up analysis, with a mean age of 22.15 ± 2.57 years. Participants were recruited through online poster advertisements for students enrolled in a college at Peking University. The inclusion criteria are shown in the Additional file 1: Appendix S1. The study was approved by the Ethics Committee of the corresponding author’s affiliated institution. All participants provided written informed consent. The study protocol was pre-registered in Open Science Framework (see osf.io/ahm6e for details).

## Materials

### Self-report scales

***The Cumulative Life Stress Inventory*** The Cumulative Life Stress Inventory for College Students was used to assess stress. It lists 35 common life stress scenarios for college students and asks respondents to assess the degree of impact and impact duration [56]. Event impact required the respondents to assess the positive or negative event: scores from − 4 to + 4 indicated an extremely severe negative impact to an extremely positive impact, respectively. Event impact duration was categorized into less than 3 months, less than 6 months, less than 1 year, and more than 1 year, and was scored from 1 to 4, respectively. The degree of impact and the duration of impact of each participant-rated negative event were multiplied to obtain the negative life event volume; all negative life event volumes were summed to obtain the total negative life events experienced. Positive life events were calculated similarly to negative life events.

***Meaning-making Scale*** Meaning-making was measured using the Meaning-making Scale, which comprised seven items on the extent that individuals constructed meaning in response to stressful events [57]. For example, “When something difficult happens, I usually understand very quickly the meaning of what is happening to me.” Items were responded to on a seven-point Likert scale, which ranged from 1 (*strongly disagree*) to 7 (*strongly agree*). In our study, the internal consistency coefficient of this scale was 0.80, indicating good internal consistency.

***Perceived Stress Scale*** Stress perception was measured using the short version of the Chinese version of the Perceived Stress Scale, which comprised 10 questions on stressful feelings of tension and loss of control [58]. For example, “In the past month, I have been upset about something unexpected happening.” Responses were rated on a five-point Likert scale, which ranged from 1 (*never*) to 5 (*always*). In our study, the internal consistency coefficient of this scale was 0.91, indicating excellent internal consistency.

***SRG Scale*** SRG was measured using the short version of the Stress-Related Growth Scale developed by Park et al. [6]. It measures positive changes in individuals after stressful events and comprised 15 items (e.g., “When I experienced stressful events, I learned to be nicer to others”). Responses were rated on a five-point scale, which ranged from 1 (*very non-compliant*) to 5 (*very compliant*) [6]. In our study, the internal consistency coefficient for this scale was 0.85, indicating good internal consistency.

***Coping Flexibility Scale*** Coping flexibility was measured using the Coping Flexibility Scale, which asked participants to evaluate whether the described coping strategy for stress in the item was applicable to them [11]. The sample item was “When a stressful situation does not improve, I will try to think of alternative ways to cope with the stress.” Responses were rated on a four-point Likert scale, which ranged from 1 (*not applicable*) to 4 (*very applicable*). In our study, the internal consistency coefficient of this scale was 0.82, indicating good internal consistency.

***Meaning in Life Scale*** The Meaning in Life Scale measures an individual’s perceived meaning in life [59]. An example was “I understand the meaning of my life.” Responses were rated on a five-point Likert scale, ranging from 1 (*strongly disagree*) to 5 (*strongly agree*). The internal consistency coefficients were 0.71 and 0.65 for mental simulation in the two conditions, respectively, indicating acceptable internal consistency.

### Experimental task

***Meaning-making Task*** We used the mental simulation paradigm [17] as the meaning-making task. It was divided into mental simulation tasks for both meaning-making and control conditions. The fMRI task-state experimental design was a block design, in which one session scan included three blocks: two experimental conditions and a distraction condition; each block of the experimental condition included three trials.

As per previous studies, we used mental simulations of future time points to initiate the meaning-making process. Compared with the present, mental simulations related to the future can initiate the meaning-making system [17]. In the experimental meaning-making condition, participants were asked to imagine two separate events that were to occur under three future conditions to prime meaning-making: 10 years later, 5 years later, and 1 year later. All three points foreshadowed a more distant time frame. There was no significant association between the two events [38]. The duration of imagination task for each trial lasted 30 s, after which a five-point scale (from 0 to 4) was assigned for the positivity, vividness, and insightfulness of the event imagery.

In the control condition, participants were asked to imagine two separate events that were to occur under three present conditions: within 24 h, in 1 h, and in 8 h. All three of these time points foreshadowed a current time frame [38]. There were no significant associations between these two events, and the duration of the imagination task was 30 s per trial. The results were evaluated similarly to the meaning-making condition.

**Fig. 3:**
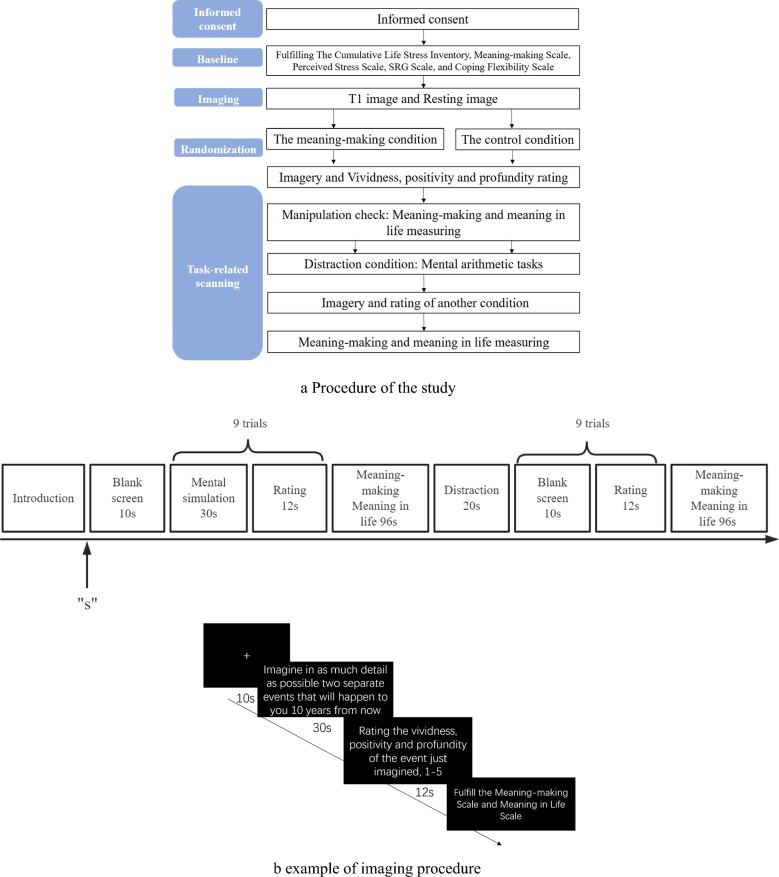
Procedure of the study and example of imaging procedure. **a** Procedure of the study. **b** example of imaging procedure

In both conditions, participants were instructed to imagine each event “with as much detail as possible.” The order of the two conditions was balanced among participants. Following this period of manipulation, participants completed measures of meaning-making and meaning in life [17]. The first 31 participants completed the imagery task with three trials per experimental condition; however, to increase result stability, the number of trials per experimental condition was increased to nine in the 32nd participant, with three repeated occurrences per trial. The distraction condition was between the experimental condition and the control condition, in which participants were asked to complete a control task that was independent of time and space: they were asked to subtract three from the presented number for 20 s. The fMRI task-state guide phrases are presented in Fig. 3a.

### Data collection procedures

Data were collected via questionnaires after the participants had signed an informed consent form. Subsequently, T1 fMRI and resting-state fMRI scans were performed. After the scans, the task-state fMRI was performed, wherein participants performed a mental simulation task. They were randomly presented with a future or present time condition, performed the imagery task as instructed, and rated the imagery content upon completion of the imagery. After the imagery and rating of the one-time condition were completed, we measured participants’ meaning-making and meaning in life and completed the manipulation check. After a distraction task, the imagery and rating of another time condition were completed, and the measures of meaning-making and meaning in life were completed again. In the end, participants were debriefed and compensated with 100 Chinese yuan (Fig. 3b).

### Scanning parameters

We collected the fMRI images for each participant using a 3T Siemens Prisma scanner and a 32-channel head coil at the Center for MRI Research at Peking University. The anatomical structure images (T1-weighted structural images, repetition time (TR) = 2530 ms; echo time (TE) = 2.98 ms; field of view (FoV) = 224 × 256 mm^2^; slice thickness = 1 mm; flip angle = 7°; voxel size = 0.5 × 0.5 × 1 mm^3^; and 192 slices) were acquired using a magnetization-prepared rapid gradient echo sequence while participants rested with their eyes closed; imaging took approximately 6 min.

Afterward, participants completed the mental simulation task and acquired functional images in the task state while using echo-planar T2* images with blood oxygenation level-dependent (BOLD) contrast. The scanning parameters were as follows: TR = 2000 ms, TE = 30 ms, FoV = 224 × 224 mm^2^, slice thickness = 2 mm, flip angle = 90°, voxel size = 2 × 2 × 2 mm^3^, and 62 slices. The acquisition sequence was interlayer scanning using a multi-band sequence.

### Statistical analysis

First, functional images were pre-processed using SPM12 (Statistical Parametric Mapping) software (the Wellcome Trust Centre for Neuroimaging, London, UK, https://www.fil.ion.ucl.ac.uk/spm/) on MATLAB platform. Next, GLM was estimated using MATLAB to obtain the activation of DMN under meaning-making. Next, we conducted correlation analysis to observe the relationship between our concerned variables using SPSS 24.0. Mediating effects were detected using SPSS 24.0. Macro Process Model 4 was used to determine whether the activation of DMN could mediate the relationship between stressful life events and SRG and its coping flexibility indicator. Finally, PPI was used to explore the FC in DMN under meaning-making using MATLAB.

### Pre-processing and analysis of fMRI data

First, we inspected the raw data. No obvious artifacts or image residuals were found. Afterward, the raw images were slice-timing corrected and aligned to the first volume to correct participants’ head motion. Head movements were corrected within each session, and we extracted six movement parameters (translation; x, y, z, and rotation; pitch, roll, yaw) for further analysis in the statistical model.

Next, we normalized the functional images to the Montreal Neurological Institute template using the parameters of anatomical normalization with a final image resolution of 2 × 2 × 2 mm^3^. They were also spatially smoothed using a 6-mm full-width at half-maximum Gaussian kernel. All fMRI images were temporally filtered using a filter high-pass with 128 s in width.

### Generalized linear modeling

In the first-level analysis, we used a block design to model the data for each participant in GLM, which served to estimate the parameters for the task effects of each voxel. The BOLD signal was convolved with the typical SPM blood flow theorem response function. We used the task type (i.e., future condition, present condition, or distractor task) and six head-motion parameters generated in the pre-processing as regressors in the model.

In the second-level analysis, we set up a future > present condition contrast and future < present condition contrast. Based on prior research that also conducted mental simulations of two independent events [17], the future condition showed a greater meaning in life than the present condition. Thus, we used the future > present condition contrast to represent the meaning-making process. Moreover, we used the activation of the brain areas in the present > future condition to represent negative activation for meaning-making (*p* < .05, FDR corrected at voxel level; extent threshold k > 64).

## Regions of interest analysis

On the basis of whole-brain activation, we further explored the activation of DMN as a seed region. We defined ROI using the Marsbar software package and obtained seven large-scale brain networks (which included the DMN) based on the organization of the human cerebral cortex through intrinsic FC [60]. Moreover, we used the data obtained on the average activation intensity of the DMN as the mask of the DMN and the activation intensity of ROI.

### Mediating effect analysis

To verify the mediating effect of meaning-making between stressful events and SRG and its indicator, coping flexibility, we established mediating models using stressful events, activation of the DMN (i.e., a cognitive neural indicator of meaning-making), and SRG and coping flexibility as the independent, mediating, and outcome variables, respectively. Four mediating models were established, including negative stressful life events ◊ activation of the DMN ◊ SRG, positive stressful life events ◊ activation of the DMN ◊ SRG, negative stressful life events ◊ activation of the DMN ◊ coping flexibility, and positive stressful life events ◊ activation of the DMN ◊ coping flexibility. A self-sampling Bootstrap procedure was taken to validate the mediation model using the SPSS 24.0 Macro Process (Preacher, Hayes, 2008). Model 4 was selected, and the number of samples was set to 5000, using 95% CIs for bias correction. The 95% CI, not including zero, indicates that the mediating effect was significant.

### Psychophysiological interaction analysis

We used the generalized form of PPI to examine the task-related FC of the DMN and focused on the meaning-making versus control conditions. The GLM model mainly included the physiological variables, psychological variables, PPI, and regressors of the six head-motion parameters. However, we focused on the PPI.

In selecting the ROI and PCC, we eventually chose the region of maximal activation in the second-order analysis as the seed region. Previous studies often selected the PCC as a core subregion of the DMN and as a seed region in PPI analysis since it is a key connectivity hub [35–37]. Hence, the region of maximal activation was the seed point (− 10 − 68 38), and we constructed a blob with a radius of 6 mm to search for regions activated in the meaning-making > control condition in the entire brain.

## Supplementary information

**Additional file 1: Appendix S1. **Inclusion criteria of participants.

## Data Availability

The dataset that support the findings of this study are available in figshare [10.6084/m9.figshare.16567050.v1].

## Abbreviations

fMRI: Functional magnetic resonance imaging
DMN: Default mode network
SRG: Stress-related growth
PPI: Psychophysiological interaction
BOLD: Blood oxygenation level-dependent
GLM: Generalized linear modeling
ROI: Regions of interest
PCC: Posterior cingulate cortex
MANOVA: Multivariate analysis of variance
mPFC: Medial prefrontal cortex
