# Supplementary material for: The mediating role of default mode network during meaning-making aroused by mental simulation between stressful events and stress-related growth: a task fMRI study
Source: Behav Brain Funct. 2023 Jul 15;19:12. doi: 10.1186/s12993-023-00214-x (PMC10350273; doi:10.1186/s12993-023-00214-x)
Supplement: Supplementary file 1 — Additional file 1: Appendix S1. Inclusion criteria of participants. [file 12993_2023_214_MOESM1_ESM.docx]

**Appendix 1 Inclusion Criteria of Participants**

The inclusion criteria were: (1) having no self-reported psychiatric disorders and normal levels of depression and anxiety; (2) no history of endocrine disorders (e.g., Cushing’s syndrome); (3) no history of other major chronic physical diseases, such as diabetes, heart disease, meningitis, severe history of brain injury (e.g., brain surgery or hemorrhage, severe head trauma, etc.); (4) taking antipsychotic, neurological, or adrenocorticotropic drugs; (5) having had no major surgery in the past 6 months; (6) having no metal implants in the body and no claustrophobia; (7) no alcohol abuse (less than/equal to 2 times per day) and no excessive smoking (less than/equal to 5 cigarettes per day); (8) no major in or double degree in Psychology; (9) being right-handed, having normal vision or corrected vision, no ophthalmic disease (students who usually wore glasses were asked to wear frame glasses, not contact lenses); (10) having no nuclear magnetic (fMRI) or brain magnetic (MEG) experiment 48 hours before the experiment; (11) no strenuous activities, no alcohol abuse, no beverages that may affect mental status (e.g., drinks containing alcohol and caffeine), and no drugs that may affect mental status (e.g., tranquilizers) taken in the 24 hours before the experiment.
